# The complete plastome sequence of Durian, *Durio zibethinus* L. (Malvaceae)

**DOI:** 10.1080/23802359.2017.1398615

**Published:** 2017-11-06

**Authors:** Se-Hwan Cheon, Sangjin Jo, Hoe-Won Kim, Young-Kee Kim, Jung-Yeon Sohn, Ki-Joong Kim

**Affiliations:** Division of Life Sciences, Korea University, Seoul, Korea

**Keywords:** Plastome, *Durio zibethinus*, durian, tropical fruit, Malvaceae

## Abstract

The complete plastome sequence of *Durio zibethinus* L. (Malvaceae) is determined in this study (NCBI acc. no. MG138151). *D. zibethinus* is an important fruit crop in Southeastern Asia and known as the ‘king of fruit’. Our *D. zibethinus* plastome is the first reported sequences from the subfamily Helicteroideae of Malvaceae. The plastome sequence of *D. zibethinus* is 163,974 bp in length and it is composed of a pair of 23,679 bp inverted repeat regions separated by large and small single-copy regions of 95,704 bp and 20,912 bp, respectively. The gene order and structure of the *D. zibethinus* are similar to those of the typical plastome of land plants. The plastome encodes 113 genes, of which 79 are protein-coding genes, 30 are tRNA genes, and four are rRNA genes. Fifteen genes contain single intron and two genes have two introns. A total of 144 simple sequence repeats (SSR) were identified in the genome. Phylogenetic analysis show that *D. zibethinus* (Helicteroideae) is sister group of *Tilia* (Tilioideae) clade with 100% bootstrap support.

*Durio zibethinus* L. is popular tropical fruit species known as the ‘king of fruit’ in Southeastern Asia (Idris 2011). The genus *Durio* consist of approximately 30 species and belonging to Helicteroideae subfamily of Malvaceae. More than 100 cultivars of *D. zibethinus* are developed during last 50 years. This study provides a reference plastome sequence for the important tropical fruit species and will be contribute to clarify the phylogenetic relationship of Malvaceae.

The leaves of *D. zibethinus* ‘Mon Thong’ were collected from Korea University greenhouse, where we grew the plants from seeds originally collected from Thailand. The voucher specimens were deposited in the Korea University Herbarium (KUS 2014-0245). Fresh leaves were ground into powder in liquid nitrogen and total genomic DNAs were extracted using the CTAB methods (Doyle and Doyle 1987). The genomic DNAs are deposited in the Plant DNA Bank in Korea (PDBK accession no. 2014-0245). The complete plastome sequences of *D. zibethinus* was generated using Illumina HiSeq 2000 system (Illumina, San Diego, CA), and assembled by Geneious version 8.1.9 (Kearse et al. 2012). The genome coverage was 866× with total 1,606,611 reads. Gene annotations were performed using BLAST from the NCBI, DOGMA (Wyman et al. 2004) and tRNAscan-SE (Lowe and Chan 2016).

The complete plastome is 163,974 bp in length, and consists of a large single copy of 95,704 bp and a small single copy of 20,912 bp, separated by two inverted repeats of 23,679 bp (NCBI acc. no. MG138151). The plastome encodes unique 113 genes (79 protein-coding genes, 30 tRNA genes, and four rRNA genes). Fifteen genes contain single intron and two genes (*clpP* and *ycf3*) have two introns. The structural organization, gene content and order, and A–T content of the plastome are similar to those of other typical Malvaceae such as *T. cacao* (Kane et al. 2012), *Gossypium* (Lee et al. 2006; Ibrahim et al. 2006; Chen et al. 2016), and other typical land plants (Kim and Lee 2004; Kim et al. 2009; Yi and Kim 2012; Jo et al. 2016). But it shows a few minor differences at the junction of inverted repeat (IR) and large single copy (LSC). Compared with the plastomes of other Malvaceae members, *rpl23* and *rpl2* genes of the *D. zibethinus* plastome are located on the LSC region instead of the IR region. A total of 144 simple sequence repeats (SSR) loci are scattered among the genome. Among these, 110, 25, and nine are mono-SSR, di-SSR, and tri-SSR loci, respectively. The locus could be useful maker to identifying the cultivars of *D. zibethinus*.

To validate the phylogenetic relationships of *D. zibethinus*, maximum-likelihood (ML) tree was constructed from 20 plastomes. The phylogenetic analysis was performed on a data set that included 79 protein-coding genes and 4 rRNA genes (aligned length: 78,812bp) from the 20 taxa using RAxML v. 7.7.1 (Stamatakis et al. 2008). Our *D. zibethinus* plastome is the first reported sequences from the subfamily Helicteroideae. The phylogenetic tree shows that *D. zibethinus* (Helicteroideae) form a monophyletic group with *Tilia* (Tilioideae) by 100 bootstrap values (Figure 1).

**Figure 1.**
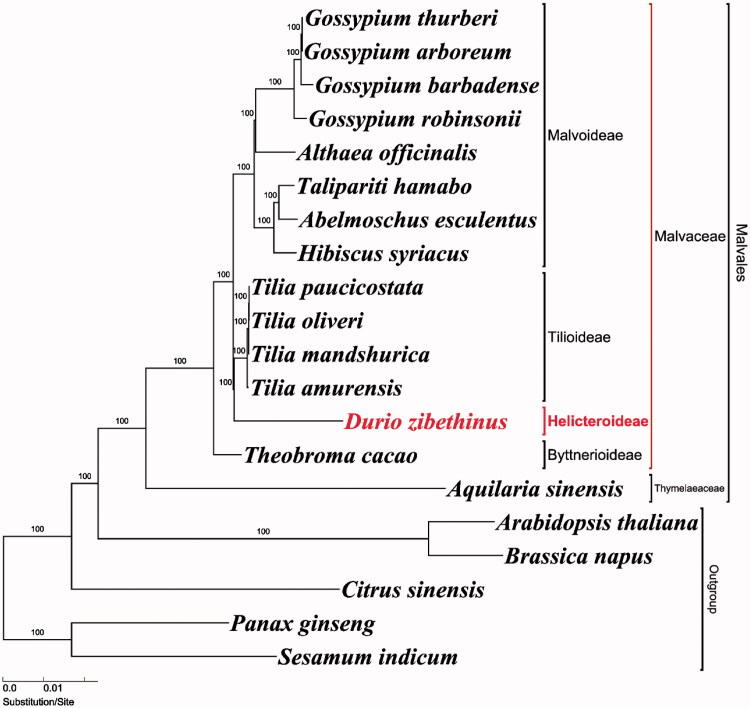
Maximum-likelihood (ML) tree based on 79 protein-coding and 4 rRNA genes from 20 plastomes as determined by RAxML (−ln L= −263,704.243992). The numbers at each node indicate the ML bootstrap values. Genbank accession numbers of taxa are shown in the following: *Abelmoschus esculentus* (NC_035234), *Althaea officinalis* (NC_034701), *Aquilaria sinensis* (NC_029243), *Arabidopsis thaliana* (NC_000932), *Brassica napus* (NC_016734), *Citrus sinensis* (NC_008334), *Durio zibethinus* (MG138151, this study), *Gossypium arboreum* (NC_016712), *Gossypium barbadense* (NC_008641), *Gossypium robinsonii* (NC_018113), *Gossypium thurberi* (NC_015204), *Hibiscus syriacus* (KR259989), *Panax ginseng* (NC_006290), *Sesamum indicum* (NC_016433), *Talipariti hamabo* (NC_030195), *Theobroma cacao* (NC_014676), *Tilia amurensis* (NC_028588), *Tilia mandshurica* (NC_028589), *Tilia oliveri* (NC_028590), and *Tilia paucicostata* (NC_028591).
